# An Assessment of the Screening Method to Evaluate Vaccine Effectiveness: The Case of 7-Valent Pneumococcal Conjugate Vaccine in the United States

**DOI:** 10.1371/journal.pone.0041785

**Published:** 2012-08-01

**Authors:** Adam L. Cohen, Thomas Taylor, Monica M. Farley, William Schaffner, Lindsey J. Lesher, Kenneth A. Gershman, Nancy M. Bennett, Arthur Reingold, Ann Thomas, Joan Baumbach, Lee H. Harrison, Susan Petit, Bernard Beall, Elizabeth Zell, Matthew Moore

**Affiliations:** 1 Centers for Disease Control and Prevention, Atlanta, Georgia, United States of America; 2 Emory University School of Medicine, Atlanta, Georgia, United States of America; 3 Department of Preventive Medicine, Vanderbilt University School of Medicine, Nashville, Tennessee, United States of America; 4 Minnesota Department of Health, St. Paul, Minnesota, United States of America; 5 Colorado Department of Health and the Environment, Denver, Colorado, United States of America; 6 Rochester University School of Medicine, Rochester, New York, United States of America; 7 University of California, Berkeley, California, United States of America; 8 Oregon Department of Health, Portland, Oregon, United States of America; 9 New Mexico Department of Health, Santa Fe, New Mexico, United States of America; 10 Johns Hopkins Bloomberg School of Public Health, Baltimore, Maryland, United States of America; 11 Connecticut Department of Public Health, Hartford, Connecticut, United States of America; Instituto Butantan, Brazil

## Abstract

The screening method, which employs readily available data, is an inexpensive and quick means of estimating vaccine effectiveness (VE). We compared estimates of effectiveness of heptavalent pneumococcal conjugate vaccine (PCV7) against invasive pneumococcal disease (IPD) using the screening and case-control methods. Cases were children aged 19–35 months with pneumococcus isolated from normally sterile sites residing in Active Bacterial Core surveillance areas in the United States. Case-control VE was estimated for 2001–2004 by comparing the odds of vaccination among cases and community controls. Screening-method VE for 2001–2009 was estimated by comparing the proportion of cases vaccinated to National Immunization Survey-derived coverage among the general population. To evaluate the plausibility of screening-method VE findings, we estimated attack rates among vaccinated and unvaccinated persons. We identified 1,154 children with IPD. Annual population PCV7 coverage with ≥1 dose increased from 38% to 97%. Case-control VE for ≥1 dose was estimated as 75% against all-serotype IPD (annual range: 35–83%) and 91% for PCV7-type IPD (annual range: 65–100%). By the screening method, the overall VE was 86% for ≥1 dose (annual range: −240–70%) against all-serotype IPD and 94% (annual range: 62–97%) against PCV7-type IPD. As cases of PCV7-type IPD declined during 2001–2005, estimated attack rates for all-serotype IPD among vaccinated and unvaccinated individuals became less consistent than what would be expected with the estimated effectiveness of PCV7. The screening method yields estimates of VE that are highly dependent on the time period during which it is used and the choice of outcome. The method should be used cautiously to evaluate VE of PCVs.

## Introduction

The World Health Organization encourages countries that introduce pneumococcal conjugate vaccine (PCV) into their routine childhood immunization system to monitor the impact of the vaccine and the vaccination program on pneumococcal disease [1]. Many countries, including the United States, administer a 3-dose infant schedule plus a booster dose in the second year of life. The United States first introduced heptavalent PCV (PCV7; Prev[e]nar, Pfizer) in 2000 which was replaced by 13-valent vaccine (PCV13) in 2010. Other countries have introduced the vaccine using other regimens, in particular, 2 doses for infants plus a booster dose or 3 infant doses without a booster.

The *efficacy* of PCV is not generally in dispute; the randomized controlled trial (RCT) remains the gold standard for calculating vaccine efficacy. A recent meta-analysis of 11 published RCTs in a number of settings found that the efficacy of a complete infant schedule of PCV7 was 80% (95% confidence interval [CI] 58–90%) against IPD caused by serotypes in PCV7 (PCV7-serotype IPD) and 58% (95% CI 29–75%) against IPD caused by any serotype (all-serotype IPD) [2]. The vaccine has demonstrated efficacy when at least 3 doses are given in the first year of life; randomized controlled trials with 2 doses in the first year of life have not been conducted.

Once vaccine has been introduced into a routine immunization program, RCTs are no longer ethical. High-quality pneumococcal surveillance can be used to assess population-level *impact*, but estimating vaccine *effectiveness*–how well the vaccine performs in the field under real world circumstances–must be done in divergent settings through observational studies, such as those using a case-control design. In contrast to RCTs which measure efficacy, observational studies can provide estimates of vaccine effectiveness. Case-control studies have limitations inherent in observational study designs, but despite that they have been quite useful in estimating vaccine effectiveness post-licensure. A large case-control study of vaccine effectiveness in the U.S. showed that one or more doses of PCV7 was highly effective (96% [95% CI 93–98%] against PCV7-serotype IPD among healthy children; 72% [95% CI 65–78%] against all-serotype IPD), and that even a single dose before eight months of age was effective against PCV7-type IPD (73% [95% CI 43–87%]) [3].

A potentially less resource-intensive technique of calculating vaccine effectiveness is the screening method [4], [5]. This method is a variant of the case-control and cohort methods where, instead of choosing one or more individual controls per case, the entire population at risk (or a representative, i.e., random, sample of the population) is used as a reference group [6]. To calculate vaccine effectiveness using this method, only three data points are needed from a given population: (1) the number of IPD cases, often available from pneumococcal surveillance, (2) the number of cases vaccinated, also often available from pneumococcal surveillance, and (3) the percent of the population vaccinated, often calculated from vaccine coverage surveys. No population-based denominator data are needed. In contrast with prospectively enrolled case-control studies and RCTs, the screening method leverages data being collected for other purposes and is, therefore, considerably less resource-intensive. This method has been proposed as a first “screening” step to determine if further evaluation is warranted [4], and it has been previously used to measure effectiveness of vaccines against *Haemophilus influenzae* type b [7], pertussis [8], measles [9], meningococcus [10], mumps [11], and influenza [12], but never, to our knowledge, PCV7. In addition, the screening method has not been validated against other methods of calculating vaccine effectiveness, such as the case-control method.

The U.S. conducts multisite, active, population-based surveillance for IPD and annual national surveys of vaccination coverage, both of which provide the data needed for the screening method [13], [14]. The IPD surveillance was used as a platform for the case-control study of PCV7 effectiveness soon after PCV7 was introduced into the U.S. [3], presenting the opportunity to compare vaccine effectiveness estimates using the screening method with those of another method. The aim of this current analysis was to compare vaccine effectiveness estimates from the screening method with those from the concurrent case-control study and the scientific literature.

## Methods

### Population Vaccine Coverage Surveys

Annual population PCV7 vaccine coverage in pneumococcal surveillance areas was estimated using the U.S. National Immunization Survey (NIS), a random-digit-dialing telephone survey of caregivers of children 19–35 months of age that is followed by mailed surveys to immunization providers as described in previous publications [15]. The NIS is sponsored by the Centers for Disease Control and Prevention (CDC) and has been conducted since 1994 to monitor vaccination coverage at the national, state, and in select local in the United States.

### Invasive Pneumococcal Disease Surveillance

To match the age group surveyed in the NIS, children who were age 19–35 months in the years from 2001 to 2009, the nine years following PCV7 introduction in the U.S. in 2000, were eligible for inclusion (e.g., eligible children 19–35 months of age in 2001 were born from February 1998 to June 2000). Cases of IPD had *Streptococcus pneumoniae* isolated from normally sterile sites and resided in one of 10 population- and laboratory-based pneumococcal Active Bacterial Core surveillance areas in the U.S., a surveillance system supported by CDC’s Emerging Infections Program Network [13]. Pneumococcal isolates were sent to reference laboratories at the Minnesota Department of Health or CDC for serotyping by the Quellung reaction and PCR [16]. Case-patients lived in California (San Francisco County, Alameda, and Contra Costa counties), Colorado (5-county Denver area), Connecticut (entire state), Georgia (20-county Atlanta metropolitan area), Maryland (Baltimore City and 5 neighboring counties [Baltimore metropolitan area]), Minnesota (Minneapolis and St. Paul in 2001–2002 and entire state beginning in 2002), New Mexico (beginning in 2004), New York (15-county Rochester and Albany areas), Oregon (3-county Portland metropolitan area), and Tennessee (Davidson, Hamilton, Knox, Shelby, and Williamson Counties). According to 2009 census estimates, the surveillance areas included 640,000 children 19–35 months of age [17].

Surveillance officers routinely contacted all clinical laboratories in their areas to identify cases of IPD and conducted periodic audits of laboratory records to ensure complete case ascertainment. Demographics, clinical course, and vaccination status of case-patients were determined through chart review using standardized forms. Recurrent cases were excluded; the first case was included. Study personnel obtained vaccination history from the primary healthcare provider or the provider where the patient reported receiving vaccinations; vaccination registries were also used in Tennessee, Georgia, Minnesota, New Mexico, and Oregon. Cases without recorded vaccination histories were excluded.

### Vaccine Effectiveness

Vaccine effectiveness is defined as the percentage reduction in the incidence of disease among vaccinated persons compared with unvaccinated persons, as given in the following equation:


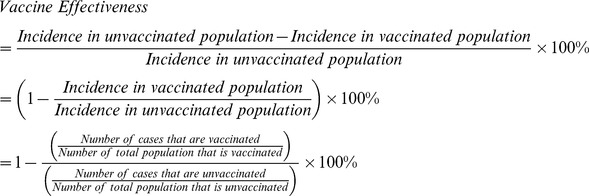


Attack rates can be substituted for incidence, but in either case, some form of population denominators are needed to calculate vaccine effectiveness. The case-control method does not require population denominators but does need controls as a comparison group. Neither is needed for the screening method, which approximates vaccine effectiveness by comparing the vaccinated proportion of children with disease with the vaccinated proportion of children in the general population [4], [5]. If we assume that the vaccinated and unvaccinated cases arise from the same population, then we can manipulate the vaccine effectiveness equation algebraically to remove the need for population denominators (i.e., the number of the total population that is vaccinated or unvaccinated) as given below, where *n* is the number of total cases and *N* is the number in the total population:


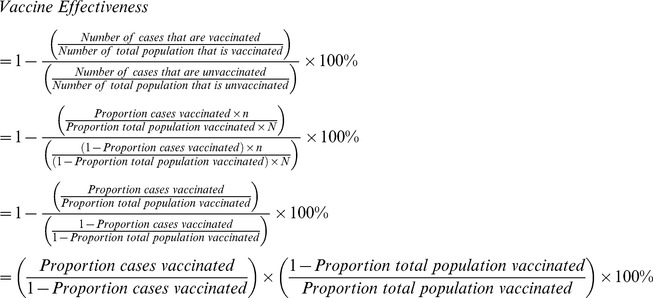


An attack rate is the number of cases in a particular population divided by the total count of that population. Since the screening method is used in situations where population denominators are not available or reliable, incidence and attack rates often cannot be calculated. However, they can be estimated using the same minimal inputs as those used for the screening method. Therefore, estimating attack rates from vaccine effectiveness estimates can be a useful way to check the validity of the vaccine effectiveness estimates; namely, attack rates should be higher among the unvaccinated population than the vaccinated if a vaccine is effective. We estimated attack rates in the following manner: The number of cases who had been vaccinated was divided by the estimated total number of people vaccinated in the population; an analogous rate was computed for non-vaccinated cases. The total vaccinated population was computed as the product of estimated coverage rates and estimated total population in the relevant geographic area; in this case, we assumed a hypothetical population of 1 million children aged 19–35 months. This hypothetical population was chosen because it is an even number and a reasonable estimate of the true population. Non-vaccinated population was the complement. These attack rates were scaled to cases per 100,000 population for convenience of dealing with integers.

Using the screening methods, we calculated the vaccine effectiveness of PCV7 against all-serotype IPD and PCV7-serotype IPD (i.e., 4, 6B, 9V, 14, 18C, 19F, and 23F) for children who received at least one PCV7 dose and separately for children who received at least 4 doses. Children that met the defined schedule were compared with children who had received no vaccination; partially vaccinated cases were excluded from the 4-dose analysis [4], [7]. We calculated annual vaccine effectiveness estimates using data from each given year and overall vaccine effectiveness estimates by pooling that data from all years together and calculated vaccine effectiveness from the pooled data.

For the matched case-control method, vaccine effectiveness was calculated using conditional logistic regression by comparing the odds of cases being vaccinated to the odds of community controls being vaccinated. We did not adjust for confounding to make this analysis comparable to the screening method analysis used here. Using previously collected data and methods from the Whitney, *et al.,* (2006) case-control study [3], we were able to calculate using a new analysis the vaccine effectiveness of the ≥1-dose schedule for all-serotype and PCV7-serotype IPD through 2004, the years of the case-control study. Due to low numbers of cases, we were unable to calculate the vaccine effectiveness of the 4-dose schedule using the case-control method; therefore, we compared the estimates from the screening method with those from the case-control methods for the ≥1-dose schedule.

### Human Subjects Review

Pneumococcal case reporting and isolate collection were considered to be public health surveillance activities exempt from CDC institutional review. All local institutional review boards at the participating surveillance sites reviewed the protocol, and approval was obtained when deemed necessary by participating sites. These sites included Emory University School of Medicine, Atlanta, Georgia; Vanderbilt University School of Medicine, Nashville, Tennessee; Minnesota Department of Health, St. Paul, Minnesota; Colorado Department of Health and the Environment, Denver, Colorado; Rochester University School of Medicine, Rochester, New York; University of California, Berkeley, Berkeley, California; Oregon Department of Health, Portland, Oregon; New Mexico Department of Health, Santa Fe, New Mexico; Johns Hopkins Bloomberg School of Public Health, Baltimore, Maryland; and Connecticut Department of Public Health, Hartford, Connecticut. Data from the National Immunization Survey is publicly available and not subject to human subjects review. Analyses using data from the PCV7 case-control study are secondary data analyses, meaning that we analyzed existing data that had been collected for another reason; human subjects review for the case-control study is discussed in the original article [3].

## Results

### Trends in Invasive Pneumococcal Disease

During the 9-year study period, 1154 children with IPD aged 19–35 months were identified; 639 (55.4%) were female, 222 (22.7%) had underlying conditions, 549 (47.7%) were hospitalized, and 17 died (1.5%). Two-thirds of the children with IPD had a known vaccine history (*n* = 779, 67.5%). Most of the IPD isolates had a known serotype (*n* = 996, 86.3%), and of those, three-quarters of the cases had a known vaccine history (*n* = 743, 74.6%). There was a steep decline in the number of all-serotype IPD cases with a known vaccine history from 103 cases in 2001 to a low of 53 cases in 2003 (Table 1). The number of IPD cases caused by serotypes included in PCV7 with a known vaccine history decreased 99% from 73 in 2001 to 1 in 2009; most of the decline was in the first few years following PCV7 introduction.

**Table 1:** Vaccine effectiveness of at least one dose and 4 or more doses of 7-valent pneumococcal conjugate vaccine (PCV7) against all-serotype invasive pneumococcal disease (IPD) and PCV7-serotype IPD among children 19–35 months of age with vaccine histories using unadjusted screening and case-control methods.

	Year									
	2001	2002	2003	2004	2005	2006	2007	2008	2009	Overall
***All-serotype IPD*** *										
Total number of cases	103	64	53	76	71	98	96	82	100	743
***≥1 doses PCV7***										
Vaccine coverage, %										
Cases	16	66	91	96	96	98	96	98	99	83
Population	38	74	90	92	95	96	98	98	97	89
Estimated attack rate, %										
Vaccinated	5	5	5	8	7	10	9	8	10	8
Unvaccinated	14	8	5	4	6	5	16	8	3	11
Vaccine effectiveness, %										
Screening method	70	33	−4	−104	−17	−98	41	2	−240	86
Case-control method	83(66, 92)	70(40,85)	71(−7, 92)	35(−261, 88)	†	†	†	†	†	75(62, 84)
***4 doses PCV7***										
Vaccine coverage, %										
Cases	2	45	76	91	91	97	95	97	99	52
Population‡	2	21	39	45	53	70	77	83	81	52
Estimated attack rate, %										
Vaccinated	6	4	2	4	3	7	7	7	8	6
Unvaccinated	9	4	3	2	4	4	13	7	3	6
Vaccine effectiveness, %										
Screening method	39	−2	19	−83	5	−98	42	7	−221	68
Case-control method	¶	¶	¶	¶	¶	¶	¶	¶	¶	¶
***PCV7-serotype IPD*** *										
Total number of cases	73	27	8	11	5	5	2	3	1	135
***≥1 dose PCV7***										
Vaccine coverage, %										
Cases	6	37	63	82	80	100	50	100	100	31
Population	38	74	90	92	95	96	98	98	97	89
Estimated attack rate, %										
Vaccinated	1	1	1	1	0	1	0	0	0	1
Unvaccinated	11	7	3	3	2	0	4	0	0	8
Vaccine effectiveness, %										
Screening method	91	79	82	62	79	′	97	′	′	94
Case-control method	95(89, 99)	84(56, 94)	100(19, 100) °	65(−489, 98)	†	†	†	†	†	91 (80, 96)
**4 doses PCV7**										
Vaccine coverage, %										
Cases	0	6	25	60	0	100	50	100	100	8
Population‡	2	21	39	45	53	70	77	83	81	52
Estimated attack rate, %										
Vaccinated	0	0	0	0	0	0	0	0	0	0
Unvaccinated	7	3	2	1	1	0	3	0	0	4
Vaccine effectiveness, %										
Screening method	100	93	92	74	100	′	97	′	′	97
Case-control method	¶	¶	¶	¶	¶	¶	¶	¶	¶	¶

* Includes only cases with vaccine history available.

† Unable to calculate because the original case-control study did not collect data on controls after 2004 [3].

‡ Partially vaccinated individuals (i.e., 1, 2, or 3 PCV7 doses) were excluded from this calculation.

¶ Unable to calculate vaccine effectiveness estimates for 4-dose schedule using the case-control method due to low numbers of cases.

°Confidence intervals calculated using Fisher’s exact test.

′Unable to calculate because of 100% vaccine coverage among case.

### Trends in Population Vaccine Coverage

The vaccine coverage among cases and in the general population increased quickly from 2001 to 2009 (Table 1). Among the general population, annual PCV7 coverage increased for the ≥1-dose schedule from 38% to 97% and for the 4-dose schedule from 2% to 81%. PCV7 coverage for both the ≥1- and 4-dose schedules was slightly lower in most years for cases of PCV7-serotype IPD compared with all-serotype IPD cases.

### Vaccine Effectiveness

Using the screening method, annual PCV7 effectiveness against all-serotype IPD was variable across years, ranging from −240% to 70% (86% overall) for the ≥1-dose schedule and from −221% to 42% (68% overall) for the 4-dose schedule (Table 1). Vaccine effectiveness against PCV7-serotype IPD was less variable (for ≥1 dose schedule, annual range from 2001 to 2009: 62–97%, 94% overall; for 4-dose schedule, annual range: 74–100.0%, 97% overall). The screening method produced vaccine effectiveness estimates for the ≥1-dose schedule that were consistently lower but similar to those using the case-control method for PCV7-serotype IPD: In three of the four years during which estimates were available for both methods, the point estimate derived from the screening method differed from the point estimate derived from the case-control method by ≤5%. For all-serotype IPD, on the other hand, estimates of vaccine effectiveness differed by >10% for all four years where we had both estimates and by >30% for three of four years (Table 1).

### Estimated Attack Rates

In general for PCV7-serotype IPD, the estimated attack rates, corresponding to the screening-method inputs, among the unvaccinated were higher than among the vaccinated (Table 1). The attack rates were more variable for all-serotype IPD, particularly after the first year or two after vaccine introduction. As cases of PCV7-type IPD declined during 2001–2005, attack rates for all-serotype IPD among vaccinated and unvaccinated individuals became more similar, while attack rates for PCV7-type IPD remained consistent with effectiveness of PCV7.

## Discussion

Is there a role for using the screening method to calculate vaccine effectiveness of PCV7? Yes, but it is a limited role. The term “screening method” was coined because this method was designed to be used as a quick, preliminary analysis when incidence and attack rate data are not available. In clinical medicine, screening tests are not expected to be diagnostic but rather to identify patients who need further evaluation for diagnosis and treatment. Similarly, vaccine effectiveness estimates derived from the screening method may require subsequent confirmation with more accurate and valid methods. Similar to diagnostic screening, a plausible result does not necessarily equate to a valid estimate of vaccine effectiveness. The computed and corresponding attack rates are an additional *de facto* screen on use of the same data inputs.

Based on results of randomized clinical trials and case-control studies, for the full-vaccine regimen and the vaccine-specific outcome PCV effectiveness estimates generally above 90% are credible. At the reduced dosage but still for the specific outcome, vaccine effectiveness estimates in the range of 80% are also credible. After the first 2–3 years and when using a non-specific endpoint such as IPD caused by all serotypes, the vaccine effectiveness estimates are highly variable and not what would be expected theoretically. We would expect vaccine effectiveness estimates to be higher against PCV7-type disease compared with all-serotype IPD. We would also expect that the complete 4-dose schedule would be more effective than receiving only 1 or more doses. Further, we would expect that the vaccine effectiveness estimates against all-serotype IPD would decline over time as fewer IPD cases are caused by serotypes included in the vaccine. These expectations were fulfilled, suggesting that the screening method may yield valid estimates of vaccine effectiveness, particularly for PCV7-type IPD during the first few years post-introduction, when our estimates were similar to those from published randomized controlled trials and comparable case-control studies [2], [3].

The estimated attack rates provide a critical opportunity to evaluate the believability of the estimates. If the screening results are to be credible, then the corresponding ARs must also be plausible. Even in the case of “good” effectiveness estimates, if the attack rates are impossible or unlikely, then the VE rates are suspect. For vaccines known by other means to be effective, such as PCV7, we would expect the attack rate to be lower in the vaccinated than the unvaccinated. The near-zero attack rate for PCV7-serotype IPD for the 4-dose schedule is what one would expect, as is the gradually declining attack rate in the unvaccinated, presumably due to herd effect. Similarly, the attack rates for PCV7-type IPD for the ≥1-dose schedule are low and gradually declining over the study period. The implausible attack rates for all-serotype IPD more than two years after vaccine introduction suggest that the screening method cannot be used reliably to estimate effectiveness of PCV7 against all-serotype IPD once the proportion of all IPD caused by PCV7 serotypes falls precipitously and non-PCV7 type disease increases.

Although the screening method simply applies the basic equation for calculating vaccine effectiveness, it is subject to biased estimates as is any secondary data analysis or observational study. However, because the screening method requires data that may be collected for other purposes, future PCV-adopting countries may consider applying this method as PCV immunization programs are rolled out worldwide. Accordingly, since we’ve shown vaccine effectiveness estimates using this method to be erratic, we submit that it is critical to understand and review the underlying assumptions and features of the method [4], [5].

**Figure 1:**
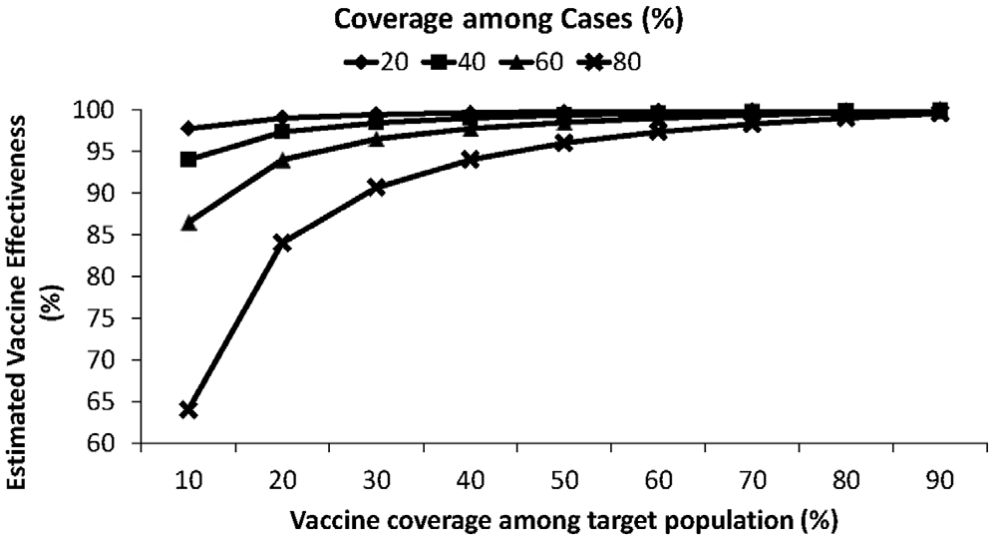
Estimated vaccine effectiveness using the screening method. This figure was created using hypothetical data created by the authors, and is adapted from a similar figure in Orenstein, et al. (1985) [4]).

Estimates of effectiveness from the screening method are very sensitive to minor errors in input estimates. For example, vaccine effectiveness will be overestimated if the population vaccine coverage is overestimated (Figure 1, adapted from [4]). For pneumococcal vaccine and other vaccines incorporated into the routine infant immunization system in the U.S. and many other countries, vaccine coverage rises quickly, which will likely adversely affect vaccine effectiveness estimates produced by the screening method 2–4 years after vaccine introduction. In addition, PCV7-serotype IPD declined dramatically after PCV7 introduction. Extrapolating from small numbers when case counts decline after vaccine introduction can lead to wide variability in vaccine effectiveness estimates. An additional contributing factor is that widespread PCV7 use leads to herd immunity, which could account for lower attack rates in the unvaccinated than otherwise expected and translate to lower estimates of vaccine effectiveness. A potential weakness of the screening method is that it may not be as reliable for evaluating vaccines that create high levels of herd immunity, such as pneumococcal in contrast to measles vaccines. We also assumed that the vaccinated and unvaccinated cases arise from the same population, which may not be the case in many developing countries.

The screening method assumes that population vaccine coverage is relatively stable and a sufficiently large proportion of the population is unvaccinated to allow the disease to remain endemic. This applies to the epidemiology of measles vaccination, a disease for which the screening method has been successfully used and for which the method was originally described [4]. However, based on our experience in the U.S., this was not the case with PCV7 coverage (which rose quickly) and IPD (which dropped soon after introduction). In addition, increases in non-vaccine serotype IPD in the years following vaccine introduction may lead to inaccurate estimates of vaccine effectiveness because such increases violate the “steady state” assumption inherent in single-point vaccine coverage or disease incidence inputs [18].

This analysis is subject to the following limitations. First, because the NIS limits its survey to children 19–35 months of age, we limited this analysis to children with IPD who were 19–35 months of age, which is slightly older than the peak age of IPD in young children [19]; vaccine effectiveness estimates may differ by age, and we were unable to calculate vaccine effectiveness for the commonly studied age group of children <5 years of age. Second, we were limited by the post-hoc case-control analysis in this manuscript, and we were unable to calculate effectiveness estimates using case-control methodology for the years 2005–2006 and for 4-dose PCV7 schedules. This also affected the number of cases with a complete vaccine history, which was only gathered reliably for patients enrolled in the case-control study and in the later years of the IPD surveillance. We limited our analysis to patients with available vaccine histories, which could bias trends shown in IPD. Third, vaccine coverage estimates should represent the populations from which the cases come. In our situation, the vaccine coverage estimates included the population at risk from the pneumococcal surveillance areas, but the sampling methodology of ABCs surveillance and the NIS precluded exactly matching populations. Fourth, we did not calculate confidence intervals for the screening method vaccine effectiveness estimates because the method uses a simple algebraic and deterministic equation. Fifth, the estimated attack rates assume a stable population, which may not be the case. Lastly, we were not able to adjust for known confounders such as underlying medical conditions of case-patients with IPD with the screening method [3]. The authors note that the overall vaccine effectiveness estimates may seem counterintuitively high compared with the annual estimates; this is a result of pooling the data overall to calculate the vaccine effectiveness and not calculating a mean of the annual estimates.

With higher valency (10- and 13-valent) pneumococcal conjugate vaccines now being introduced in many countries worldwide, there will be an increased need for evaluations of the impact and effectiveness of pneumococcal conjugate vaccine. The screening method should be used cautiously to evaluate VE of PCVs. Those using the screening method should remember that small differences in the data due to poor quality surveillance can lead to large differences in vaccine effectiveness estimates. Other less resource-intensive methods, such as the indirect cohort method, have been used successfully; however, it requires serotyping of pneumococcal isolates, cannot estimate vaccine effectiveness against all-serotype IPD, and may not be able to account for herd immunity [20]. In summary, the screening method should only be used as a preliminary test in situations where the data inputs are valid, more reliable evaluations of vaccine impact are not feasible, where sufficient cases are available, and vaccine coverage has not peaked in the general population.
